# Modeling the Interplay of Time, Space, Users, Topics, and Sentiment: A Novel Analytical Framework for Social Media Data Analysis

**DOI:** 10.3390/bs16101876

**Published:** 2026-10-12

**Authors:** Wenjing Gui, Xuefan Dong, Chen Wang, Shuai Chen, Wei Zhang

**Affiliations:** 1 School of Journalism, Communication University of China, No. 1 Dingfuzhuang East Street, Beijing 100024, China; wenjinggui@cuc.edu.cn; 2 College of Economics and Management, Beijing University of Technology, Beijing 100124, China; dongxf@bjut.edu.cn (X.D.); junowang@emails.bjut.edu.cn (C.W.); chenshuai123@emails.bjut.edu.cn (S.C.); 3 College of Marxism, Xinjiang Normal University, No. 100 Guanjing Road, Shuimogou District, Urumqi 830017, China

**Keywords:** social media data, analytical framework, cross-analysis, public perception, corporate reputation, crisis communication

## Abstract

This study addresses the gap in existing social media discourse research, which often treats time, space, users, topics, and sentiment in isolation. It proposes an integrated analytical framework to capture the interplay among these five dimensions, using an online controversy involving Nongfu Spring as an illustrative case to demonstrate the framework’s applicability. A multi-method approach combines temporal, spatial, and user classification analysis; deep learning-based sentiment analysis via a fine-tuned Chinese-BERT-wwm-ext model; and influential keyword identification using Graph Attention Network and improved TextRank algorithms. The framework is applied to 12,674 Weibo posts related to this controversy. Posts clustered temporally around key discussion periods, spatially concentrated in economically developed regions, and, while ordinary users produced the majority of posts, verified institutional and high-influence individual accounts became increasingly prominent in the later phases of the discussion. The integrated analysis reveals joint patterns across dimensions, such as the marked shift in user composition over the course of the crisis, that single-dimension analyses alone would not reveal. The study contributes a reusable framework for the multidimensional analysis of online discourse and illustrates its applicability through a single case; its generalizability across platforms and event types remains to be tested in future research.

## 1. Introduction

Social media data represents a primary form of user-generated content, arising from interactions within digital platforms (Dong & Wu, 2015), which have evolved into spaces for communication, bringing together individuals from varied backgrounds and enabling diverse interactions. Leveraging social media data for academic research presents several notable advantages. First, it is cost-effective; unlike traditional methods like surveys or interviews, which are both resource-intensive and time-consuming, social media data is readily accessible, often free, and reduces the need for extensive resources. Second, it overcomes temporal and spatial limitations. Temporal advantages include access to consistently recorded data over time, supporting longitudinal and comparative analyses. Spatially, social media data enables geographically diverse research, facilitating cross-national studies. Third, this data is inherently diverse, encompassing a broad range of users from different professions, backgrounds, and interests. Finally, social media data can be specifically applied to targeted research objectives, such as evaluating governmental policies or gauging public opinion on political events. For example, platforms like Weibo in China and Twitter in the United States have proven valuable in analyzing public sentiment during significant events, including disasters (Z. Han et al., 2022) and elections (Bovet & Makse, 2019).

In the study of online communication, understanding the various elements that shape these associations is essential (Felix et al., 2017; Yuksel et al., 2016). Social media platforms function as dynamic environments where discussions are shaped by a complex interplay of factors. The joint configuration of time, space, users, topics, and sentiments captures the complex nature of online conversations. Specifically, the temporal dimension encompasses patterns and trends influencing user activity and the dissemination of content (Chua et al., 2016). Spatial elements add a geographic layer, revealing regional variations in user behavior and cultural preferences (Xiao et al., 2022). User demographics, interests, and emotions further define the context of these discussions (Denis-Robichaud et al., 2024). Topics serve as focal points around which conversations coalesce (Ma et al., 2024; Nakagawa et al., 2022), encompassing a broad spectrum from global events to niche interests, thereby driving engagement and shaping online narratives. Sentiments contribute an emotional dimension, reflecting user attitudes and responses to diverse issues (He & Sun, 2024).

Table 1 illustrates several existing research frameworks for social media data analysis. As Table 1 shows, the design of these frameworks generally follows a methodical approach tailored to specific research or operational objectives. A common feature among many frameworks is the use of APIs for data collection, enabling systematic extraction from social media platforms to ensure datasets accurately capture user interactions. After data collection, pre-processing steps such as filtering and cleaning are crucial to eliminate noise and prepare data for analysis, thereby enhancing the accuracy and reliability of results. Analytical techniques including content analysis, sentiment analysis, trend analysis, and social network analysis are frequently employed. Each technique yields unique insights: content analysis identifies themes and patterns, sentiment analysis assesses the emotional tone of discussions, trend analysis tracks topic evolution over time, and social network analysis uncovers the structure and dynamics of user interactions.

Innovations within these frameworks often arise from their application to specific contexts or challenges. For instance, frameworks designed for analyzing political communication focus on identifying and tracking politically relevant content to gauge public sentiment and its implications for elections or policy debates (Simonofski et al., 2021). Similarly, frameworks targeting brand management emphasize consumer sentiment analysis and engagement metrics to inform marketing strategies and strengthen customer relations (Sivarajah et al., 2020). Given the global reach of social media, another critical innovation is cross-language data processing, which facilitates content analysis across multiple languages, thus broadening the applicability of social media analytics beyond linguistic boundaries (Levi et al., 2024). These frameworks contribute significantly to the literature by providing structured methodologies that can be replicated and adapted across different research contexts. They also establish a foundation for empirical research, enabling systematic assessments of social media impacts across domains such as politics, marketing, and public health.

However, existing frameworks frequently overlook the interrelationships among various data types that could yield more valuable insights, often treating social media data elements as isolated variables. For example, sentiment analysis frameworks may focus solely on evaluating the emotional tone of posts, without considering how sentiments are associated with other elements, such as network structures or temporal trends (Hamdi et al., 2023). This compartmentalized approach misses potential insights from analyzing interdependencies, such as correlations between sentiment shifts and emerging network clusters or the influence of specific events on public opinion over time. Additionally, there is a notable shortage of frameworks that support a holistic, multidimensional analysis, which would cohesively integrate diverse data elements into a unified analytical model.

This research, therefore, introduces a novel analytical framework designed to conduct a multidimensional analysis of social media data, integrating elements such as textual content, user demographics, and temporal patterns. The primary objective is to develop a framework that supports systematic cross-analysis of these elements and to illustrate its applicability in an empirical case. The framework is descriptive in nature: it identifies how dimensions are jointly distributed and how these joint distributions evolve, rather than estimating causal or statistical interaction effects.

The contribution of this study is as follows. First, whereas existing frameworks typically examine social media dimensions separately or in ad hoc combinations, the proposed framework systematizes the full set of pairwise and three-dimensional combinations among time, space, users, topics, and sentiment, and couples this inventory with a screening procedure in which preliminary statistical tests indicate which cross-analyses merit in-depth interpretation. Second, the framework is accompanied by a concrete and reusable analytical pipeline that integrates dimension extraction, deep learning-based sentiment classification, and graph-based keyword identification. Third, the case study below serves as an illustration of applicability: it demonstrates step by step how the framework is operationalized, and it shows what the integrated analyses add beyond single-dimension descriptions of the same data.

## 2. The Proposed General Framework

### 2.1. Cross-Analysis Between Two Elements

Table 2 presents a matrix outlining the analytical possibilities arising from pairwise combinations of five key elements of social media data: time, space, users, topics, and sentiments. Each intersection illustrates how the integration of two elements can uncover distinct patterns or dynamics. For instance, the time–space dimension enables the identification of temporal engagement patterns across regions, such as peak activity during local events (S. Li et al., 2020). Time–user analysis facilitates the examination of user behavior trends over time (Kang, 2022; Lian et al., 2024), while time–topic and time–sentiment pairings support the study of topic evolution and emotional fluctuations across specific periods (Pathak et al., 2021; Sukhwal & Kankanhalli, 2022). Pairings of space with users (Hwong et al., 2017), topics (Shah & Househ, 2024), and sentiments (Paolanti et al., 2021) reveal regional heterogeneity in user demographics, content preferences, and emotional responses. User–topic (Zhan et al., 2017) and user–sentiment (Wilk et al., 2023) intersections allow for the segmentation of user groups based on interests and affective tendencies. Finally, topic–sentiment analysis captures how emotional expressions vary across different thematic discussions (Lyu et al., 2021).

### 2.2. Cross-Analysis Between Three Elements

Expanding on the pairwise analysis of social media data elements, examining intersections among three elements reveals additional insights into how temporal, spatial, and user dimensions intersect. This multi-dimensional analysis helps researchers understand how time, location, user characteristics, topics, and sentiment are jointly distributed. Such insights can inform decision-making, guide context-sensitive communication, and improve the effectiveness of social media engagement across different audiences and regions.

#### 2.2.1. Time, Space, and User

This intersection involves studying how user behavior, such as posting frequency and content preferences, vary across different timeframes and geographic regions (Wilkins et al., 2021). For example, during natural disasters like hurricanes or earthquakes, user engagement often peaks in affected regions at specific times as the event unfolds and emergency responses are coordinated. Understanding these patterns enables organizations to tailor content distribution according to regional needs and activity cycles, such as posting essential updates during peak local engagement hours. This approach allows for responsive and effective communication that aligns with both regional demands and user engagement rhythms over time.

#### 2.2.2. Time, Space, and Topic

Analyzing the intersection of time, space, and topic can reveal regional and temporal trends in discussion themes, such as how specific topics gain attention across different regions and time periods (Levi et al., 2024). For instance, during major sports events like the World Cup, interest in team performance, scores, and player statistics can vary widely depending on local time zones and regional team affiliations. This intersection enables content creators and marketers to develop geographically targeted campaigns that align with local interests and timeframes, enhancing engagement by delivering relevant content as regional enthusiasm peaks. Additionally, tracking topic shifts across regions and time provides insights into broader cultural or international trends, which can guide long-term campaign planning.

#### 2.2.3. Time, Space, and Sentiment

Exploring time, space, and sentiment together offers insights into how public emotions fluctuate across regions and time in response to major events or policy changes (S. Wang et al., 2024). For example, during a global health crisis such as the COVID-19 pandemic, regional variations in sentiment could reflect responses to government restrictions, local case surges, or vaccine rollouts, with sentiment often shifting over time as these factors evolve. Identifying regional emotional patterns enables health organizations and policymakers to adjust communication strategies, issuing supportive messages in regions experiencing high anxiety or providing targeted information during periods of peak emotional response. This layered understanding aids in addressing local concerns effectively, fostering public trust, and managing crises with sensitivity to both regional and temporal sentiment variations (Morgan & Wilk, 2021; Munaf et al., 2023).

#### 2.2.4. Time, User, and Topic

This intersection helps in understanding how specific topics engage different user groups over time, revealing shifts in user interest and behavior (Avalle et al., 2024). For instance, a brand might observe that sustainability topics initially resonate strongly with younger demographics, with engagement peaking during environmental awareness months. However, over time, engagement may grow among broader age groups as awareness spreads and public conversations evolve. Recognizing these dynamics allows brands to develop evolving content strategies that keep pace with changing audience interests, targeting different demographics with tailored messages as topics gain relevance. This approach is particularly valuable for ongoing social issues, as it supports sustained engagement across diverse user groups.

#### 2.2.5. Time, User, and Sentiment

Analyzing the intersection of time, user, and sentiment reveals how emotional responses vary among user groups over specific period (Lanier et al., 2022; Morgan & Wilk, 2021). For example, in response to new technology releases, tech-savvy users may express excitement early on, while less engaged users might show increased interest or adopt a wait-and-see attitude over time. Brands can use these insights to adapt their messaging by targeting early adopters with technical specifications and reviews and then shifting to more user-friendly content as the general public becomes more interested. Tracking sentiment trends across user segments enables companies to align their engagement strategies with the evolving emotional climate of distinct user groups, supporting responsive marketing that adapts to sentiment shifts.

#### 2.2.6. Time, Topic, and Sentiment

Analyzing time, topic, and sentiment involves understanding how sentiment towards specific topics fluctuates over time (Park & Suh, 2023). Exploring the temporal dynamics of sentiment associated with different topics provides insights into public reactions and emotional responses (Aldosery et al., 2024; X. Li et al., 2023). For example, during the rollout of a controversial policy, public sentiment may initially be negative, but as time passes and more information is shared, opinions might shift or stabilize. Tracking these sentiment trends in relation to key topics and time helps policymakers and organizations gauge public reaction and modify communication strategies to address prevailing concerns. Such analysis is also crucial for reputation management, allowing businesses to monitor sentiment around product launches or service issues, adjust responses based on feedback, and shape their public image in line with evolving sentiment dynamics around relevant topics.

#### 2.2.7. Space, User, and Topic

The intersection of space, user, and topic examines how user demographics in specific regions engage with particular subjects, providing insights into geographically driven preferences and behaviors (Jha & Verma, 2024; Y. Wang, 2024). This approach helps clarify how local contexts shape topic relevance and discussion among user groups. For instance, health-related topics might see greater engagement with fitness trends in urban areas with younger populations, while rural areas may focus more on healthcare access. Identifying key users in these regions can also reveal how local discourse is shaped. This knowledge supports the development of targeted, region-specific content that aligns with local demographic characteristics and interests.

#### 2.2.8. Space, User, and Sentiment

Analyzing space, user, and sentiment highlights how emotional responses vary by geographic and demographic segments, capturing cultural differences in attitudes toward certain topics or events (Zhang et al., 2022). For example, a global event on climate policy may receive strong support in regions with a history of environmental activism, while it might prompt neutral or skeptical reactions in areas reliant on fossil fuel industries. Recognizing these variations allows for tailored messaging strategies that resonate with local attitudes and sentiments. This approach supports more effective engagement, as messages can be adapted to reflect specific regional and demographic perspectives.

#### 2.2.9. Space, Topic, and Sentiment

The intersection of space, topic, and sentiment captures regional variations in public emotions tied to specific topics, helping to clarify how cultural and social factors influence attitudes across geographic boundaries (Bashar et al., 2022). For instance, discussions around renewable energy policies may generate positive sentiment in areas focused on sustainability but mixed responses in regions with economies linked to traditional energy sources. Mapping these sentiment patterns aids in developing communication strategies that respect regional views. This type of analysis is valuable for organizations aiming to engage regional audiences in ways that reflect both local interests and emotional contexts.

#### 2.2.10. User, Topic, and Sentiment

Exploring the intersection of user, topic, and sentiment reveals how different user demographics express emotions toward specific topics, showing sentiment patterns that reflect the perspectives of various audience segments. For instance, a tech company launching a new product may find that younger users express enthusiasm, while older users might have concerns about usability or security. Tailoring communication strategies to align with each group’s emotional responses can improve engagement and address concerns effectively. This type of targeted approach supports audience segmentation based on sentiment, enhancing the relevance of content for each demographic.

## 3. Case Study

### 3.1. Data

On 25 February 2024, the death of Wahaha founder Zong Qinghou was followed by extensive online tributes. During the same period, a subset of Weibo posts discussed Nongfu Spring and its founder, Zhong Shanshan. Some posts questioned Zhong’s absence from Zong’s funeral, while others described Nongfu Spring’s packaging as having a “Japanese-style” aesthetic. After Zhong issued clarifications, related discussion continued, and some posts used terms such as “boycott” or “unpatriotic.” Within the sampled discourse, these terms indicate how some social media users framed the event; they should not be read as the authors’ assessment of either company or its founder. Because this study analyzes social media data only, it does not evaluate the factual accuracy of these user claims, actual corporate conduct, or any market or financial consequences of the event.

This case was selected because it aligns with the study’s framework, which is designed for analyzing social media data through the intersection of five elements: time, space, user, topic, and sentiment. The case contains temporal, geographic, user, topical, and sentiment variation within a bounded discussion period, making it suitable for demonstrating the framework’s operation. It also provides an empirical setting for examining how identity-related expressions co-occurred with discussions of corporate reputation in sampled Weibo posts. The analysis does not treat such expressions as evidence of users’ stable political attitudes, nor does it assess the actual conduct of the companies involved. Instead, it examines how topics, sentiments, user categories, time, and displayed locations were jointly distributed during the discussion.

The data for this analysis was sourced from Weibo, a major social media platform in China, capturing discussions from 25 February 2024 to 30 April 2024. The data collection focused on posts containing specific keywords such as “Wahaha” and “Nongfu Spring” which were central to public discourse on this issue. To collect relevant data, we employed Octopus, a web extraction tool that efficiently fetched key data points, targeting specific content associated with public discourse on these keywords (Pu et al., 2022). Data were collected in two waves: an initial collection covered 25 February to 10 March 2024 (the acute phase of the controversy) and was subsequently extended to 30 April 2024, so that the dataset would cover the full evolution of the crisis. All analyses reported below refer to this complete observation window (25 February–30 April 2024). Following data collection, an extensive preprocessing stage was undertaken to ensure data quality and relevance. The retained fields (post content, posting time, displayed location, and user verification status) are textual or categorical; therefore, no numerical imputation was applied, and records lacking post content or a valid timestamp were removed rather than imputed. The preprocessing steps comprised the removal of exact duplicate records (identical combinations of content, user, and timestamp), the filtering of irrelevant content such as advertisements and spam, and the standardization of timestamp and text formats. Additionally, removing stop words (e.g., “的,” “了,” “是”) enhanced sentiment analysis and keyword extraction. These steps yielded a final dataset of 12,674 posts for analysis; Table 3 summarizes its composition.

Because the triggering event was the death of Wahaha’s founder, the dataset contains two partially overlapping discourse streams: tributes to Zong Qinghou and Wahaha on the one hand, and criticism of Nongfu Spring on the other. To gauge their relative weight, post content was screened with keyword rules: posts that referenced Wahaha or Zong Qinghou, or contained condolence expressions (e.g., “一路走好”), but did not mention Nongfu Spring were classified as tribute-oriented. This screening identified 1334 tribute-oriented posts (10.5% of the dataset). These posts were retained because the tribute discourse formed the immediate context in which the crisis unfolded. As a robustness check, however, the temporal and spatial distributions reported below were recomputed after excluding these posts; the peak activity day (3 March 2024) and the ranking of regions by post volume were unchanged, indicating that the main findings are not driven by the tribute stream. The coexistence of the two streams is further acknowledged as a limitation in Section 5.

### 3.2. Methods

#### 3.2.1. Temporal, Geographical and User Characteristics

In this study, the temporal aspect was derived from the posting time of each post. For the location dimension, the study relied on the displayed IP territory shown on each post. Since April 2022, Weibo mandatorily displays the IP territory of each post, and users cannot disable this display; the displayed IP territory is therefore a platform-recorded attribute rather than a self-reported one. This choice aligns with Weibo’s policies, which prioritize the location information visible to the public, and ensures that the analysis reflects the context in which the posts were made; it also enhances reliability by focusing on observable attributes rather than user-provided information, which may be subject to privacy settings or inaccuracies. It should be emphasized that the displayed IP territory is a coarse-grained indicator, province-level within mainland China and country- or region-level for posts from abroad, that reflects the network access point from which a post was made. It does not constitute an exact geolocation of the user, and it may diverge from the user’s place of residence (for example, when posts are made while travelling or through proxy networks). Accordingly, we use the terms “displayed IP territory” and “displayed location” throughout this paper, and all spatial findings describe the distribution of displayed locations rather than the verified geography of users. All 12,674 posts carried a displayed location. Following the four-region scheme of the National Bureau of Statistics of China, mainland provinces were grouped into East (Beijing, Tianjin, Hebei, Shanghai, Jiangsu, Zhejiang, Fujian, Shandong, Guangdong, and Hainan), Central (Shanxi, Anhui, Jiangxi, Henan, Hubei, and Hunan), West (Inner Mongolia, Guangxi, Chongqing, Sichuan, Guizhou, Yunnan, Tibet, Shaanxi, Gansu, Qinghai, Ningxia, and Xinjiang), and Northeast (Liaoning, Jilin, and Heilongjiang). Posts originating from foreign countries were grouped as Overseas. The resulting regional distribution is reported in Table 3.

For cross-analyses involving time, the observation window was divided into four phases of approximately equal length (14–20 days): Phase 1 (25 February–15 March), Phase 2 (16 March–1 April), Phase 3 (2–15 April), and Phase 4 (16–30 April). The cut-off dates combine the observed posting dynamics with identifiable developments in the controversy rather than reflecting an arbitrary analytical decision. Phase 1 covers the acute stage of the controversy: it contains 87.0% of all sampled posts, including the daily peak on 3 March 2024, shortly after the founder of Nongfu Spring issued public clarifications regarding the controversy. Additionally, 15 March marks the end of this acute stage—daily posting volume dropped by more than an order of magnitude thereafter—and mid-March also coincides with China’s Consumer Rights Day (15 March), after which media and public attention to consumer-brand controversies typically recedes. Furthermore, 1 April separates the late-March discussion, which turned toward market- and leadership-related topics (Section 4.2), from a quiet interval in early April (Phase 3 accounts for only 1.2% of all posts), and 15 April precedes the renewed increase in posting activity in late April, when widely shared posts and media coverage brought the controversy back to public attention (Section 4.1). The phase boundaries are thus anchored in posting-volume turning points and identifiable developments in the controversy, and the near-equal window lengths facilitate comparison across phases.

User information was categorized based on Weibo’s official user classification system, which includes ordinary users, Vblue, Vyellow, Vorange, and Vgold categories. Each classification reflects varying levels of verified identity and influence on the Weibo platform, making them particularly relevant for analyzing user impact in corporate reputation crises. Ordinary users lack verification, while Vblue is assigned to verified institutional accounts, such as businesses, media outlets, and government agencies, indicating official organizational presence. Vyellow applies to individuals whose identity has been authenticated by Weibo, signaling verified personal accounts. Vorange and Vgold are distinctions given to individuals with substantial influence within specific fields, meeting criteria such as follower count, engagement metrics, and readership in the past 30 days, with Vgold representing a higher influence tier. This classification system is integral to our research framework, as it allows us to explore the roles of users with different credibility levels in shaping public sentiment and engagement. In the present dataset, ordinary users account for 10,883 posts (85.9%), Vyellow for 1122 (8.8%), Vgold for 328 (2.6%), Vorange for 300 (2.4%), and Vblue for 41 (0.3%).

#### 3.2.2. Sentiment Analysis

In this study, sentiment analysis was conducted by utilizing Chinese-BERT-wwm-ext, a deep learning-based model pre-trained specifically for the Chinese language (Cui et al., 2021). As part of the BERT (Bidirectional Encoder Representations from Transformers) family, this model has demonstrated strong performance in Chinese natural language processing tasks, particularly sentiment classification. Chinese-BERT-wwm-ext was fine-tuned on the SMP2020-EWECT dataset, which is widely recognized for its comprehensive coverage of emotions in Chinese social media contexts (G. Chen et al., 2022; S. Wang et al., 2024). SMP2020-EWECT comprises 34,768 manually annotated Weibo posts; following common practice, the original emotion labels were mapped to a binary polarity scheme (positive vs. negative). The dataset was randomly divided into training (80%), validation (10%), and test (10%) subsets. Fine-tuning employed the AdamW optimizer with a learning rate of 2 × 10^−5^, a batch size of 32, and a maximum sequence length of 128 tokens, for up to five epochs; the checkpoint with the highest macro-F1 on the validation subset was retained. On the held-out test subset, the fine-tuned model achieved an accuracy of 0.81 and a macro-F1 of 0.77. Specifically, the fine-tuned Chinese-BERT-wwm-ext model processes Weibo posts as input and generates sentiment scores ranging from −0.5 to 0.5, with scores above 0 indicating positive sentiment and scores below 0 indicating negative sentiment. To validate the model on the specific corpus analyzed here, two annotators independently coded a random subsample of 300 posts as positive or negative (percentage agreement 88%, Cohen’s κ = 0.78); disagreements were resolved through discussion. Against these human annotations, the model achieved an accuracy of 0.79 and a macro-F1 of 0.76, indicating acceptable transfer to the case corpus.

#### 3.2.3. Influential Keyword Identification

In this study, opinion analysis employed the Graph Attention Network (GAT) algorithm and TextRank algorithm to extract significant keywords from the dataset and generate a word cloud (Ren et al., 2023). First, we performed word segmentation to form a word co-occurrence network, representing each document’s nodes as keywords and edges as their co-occurrence frequency. For the GAT model, a feature matrix of word vectors is generated using each word’s positional, frequency, and co-occurrence characteristics. In the attention layer, the attention coefficient *e_ij_* between nodes *i* and *j* is calculated by concatenating their feature vectors and applying a shared parameter matrix to yield attention coefficients. Parameters in the GAT model include the LeakyReLU activation function with a negative slope of 0.2, a learning rate of 0.005, and an epoch count of 500 for convergence. Attention is normalized using the softmax function, transforming the feature matrix into a final node representation enriched with contextual information. The improved TextRank algorithm is employed in the keyword extraction layer, where a probability transfer matrix is used to score node importance. The Euclidean distance matrix represents the information differences, and high-scoring nodes are selected as keywords for the word cloud, highlighting dominant discourse terms. To evaluate extraction quality, the pipeline was compared against two standard baselines, namely the original TextRank algorithm and TF–IDF. For 100 randomly sampled posts, two annotators manually identified the salient keywords as a reference; the GAT–improved TextRank pipeline achieved a Precision@20 of 0.703, compared with 0.669 for TextRank and 0.547 for TF–IDF.

#### 3.2.4. Statistical Analysis

To avoid an arbitrary selection of cross-analyses and to substantiate the differences described in the Section 4, chi-square tests were computed for all pairwise intersections among the four categorical dimensions (time, space, user, and sentiment); topics, being open-ended keywords rather than a closed set of categories, cannot be tabulated and are excluded from this screening. A pairwise cross-analysis is interpreted in detail only when the corresponding test reaches significance (*p* < 0.05). Table 4 summarizes the results. It should be emphasized that these tests establish associations among dimensions rather than causal or statistical interaction effects.

Before interpreting these tests, we examined their expected-frequency assumptions. For the Phase × Region table, only one of 20 cells (5%) had an expected count below 5 (Phase 3 × Overseas, expected count = 2.6), and no expected count was below 1, satisfying Cochran’s criterion; no expected count in the Phase × Sentiment, Region × Sentiment, or User × Sentiment tables fell below 5. For the two tests involving user categories, however, the small Vblue stratum and, in the regional analysis, the small Overseas stratum produced cells with low expected frequencies, so the asymptotic chi-square approximation may be unreliable. These two tests were therefore re-evaluated using a Monte Carlo procedure: 20,000 contingency tables with the observed margins were generated at random under the null hypothesis of independence, and each *p*-value was computed as the proportion of simulated chi-square statistics at least as large as the observed value. The Monte Carlo results confirmed both associations (Phase × User: *p* < 0.001; Region × User: *p* = 0.002), indicating that the conclusions drawn from these tests do not depend on the asymptotic approximation.

## 4. Results

### 4.1. Analysis Results for Time, Space, and User Categories

**Figure 1:**
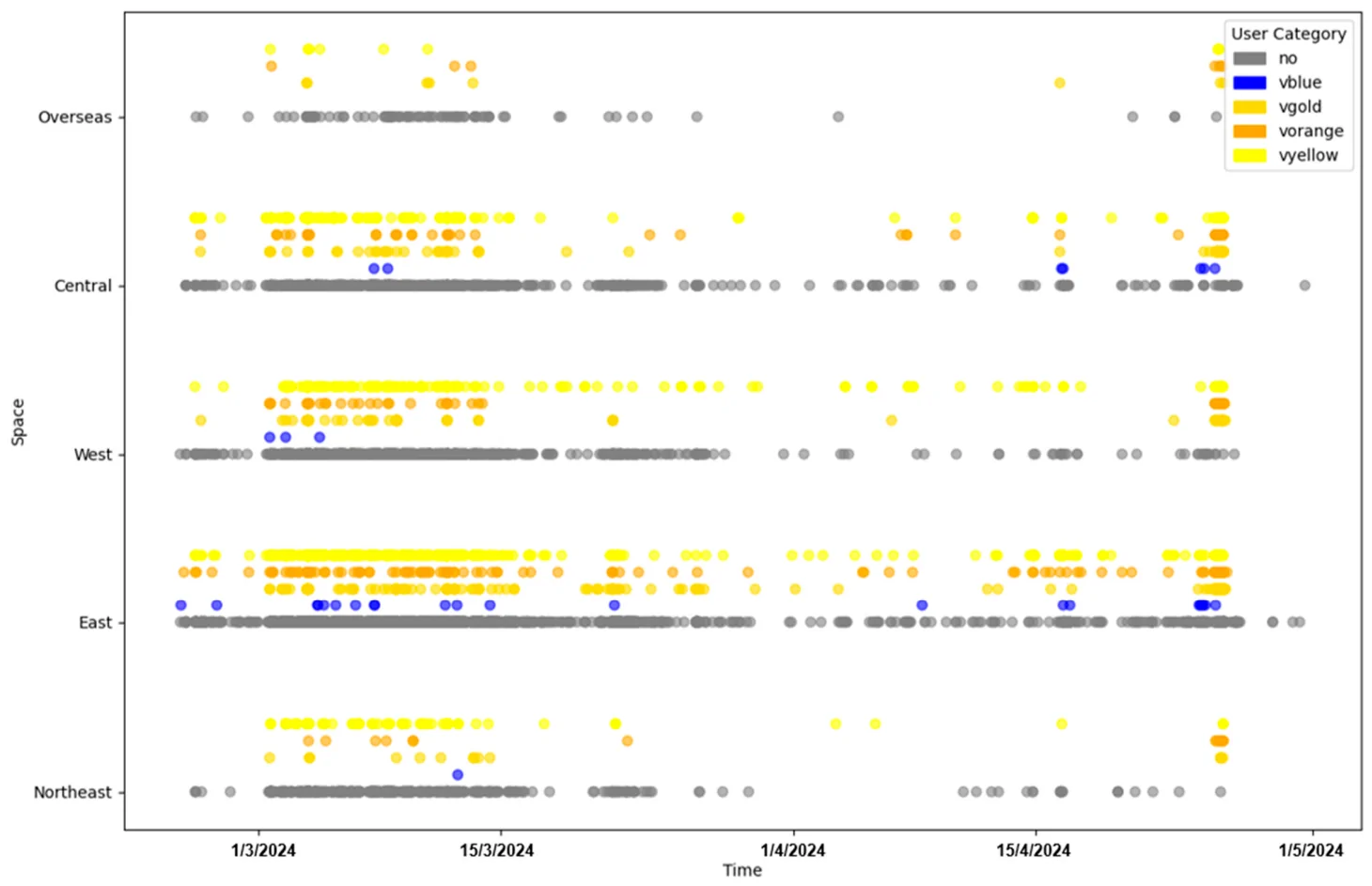
Cross-analysis results between time, space and user.

Figure 1 examines three dimensions—time, space, and user category—with distinct colors for user categories and scattered data points for the regions over the period from 25 February 2024 to 30 April 2024. Temporal spikes in mid-March and late April coincide with key developments in the controversy, such as widely shared posts and media coverage. Spatially, the East and Central regions account for the largest share of posts; both companies are headquartered in Hangzhou (eastern China), which may partly account for this concentration, although the present data do not allow this explanation to be tested. The distribution of user categories adds a further layer: Vblue (institutional) accounts are relatively sparse but appear at salient moments, a pattern consistent with institutional actors intervening when attention peaks, although their communicative intent cannot be determined from our data. Verified individual users (Vyellow, Vorange, and Vgold) are concentrated in the East and Central regions during peak periods, whereas ordinary users dominate numerically across all regions, particularly in the Overseas segment. The chi-square tests reported in Table 4 indicate that most of these descriptive patterns reflect statistically significant associations: user composition shifted markedly across phases (χ^2^(12) = 2006.45, *p* < 0.001, Cramér’s V = 0.230), with the share of verified accounts rising from 11.3% in Phase 1 to 55.2% in Phase 4. By contrast, the Phase × Region association, although significant, was very small in magnitude (χ^2^(12) = 49.41, *p* < 0.001, Cramér’s V = 0.036), indicating that regional profiles are broadly similar across phases. Because the sample is large (N = 12,674), even substantively negligible departures from independence reach conventional significance levels, so the Phase × Region result should not be read as evidence of meaningful regional shifts over time.

### 4.2. Analysis Results for Time, Space, and Topic Categories

**Figure 2:**
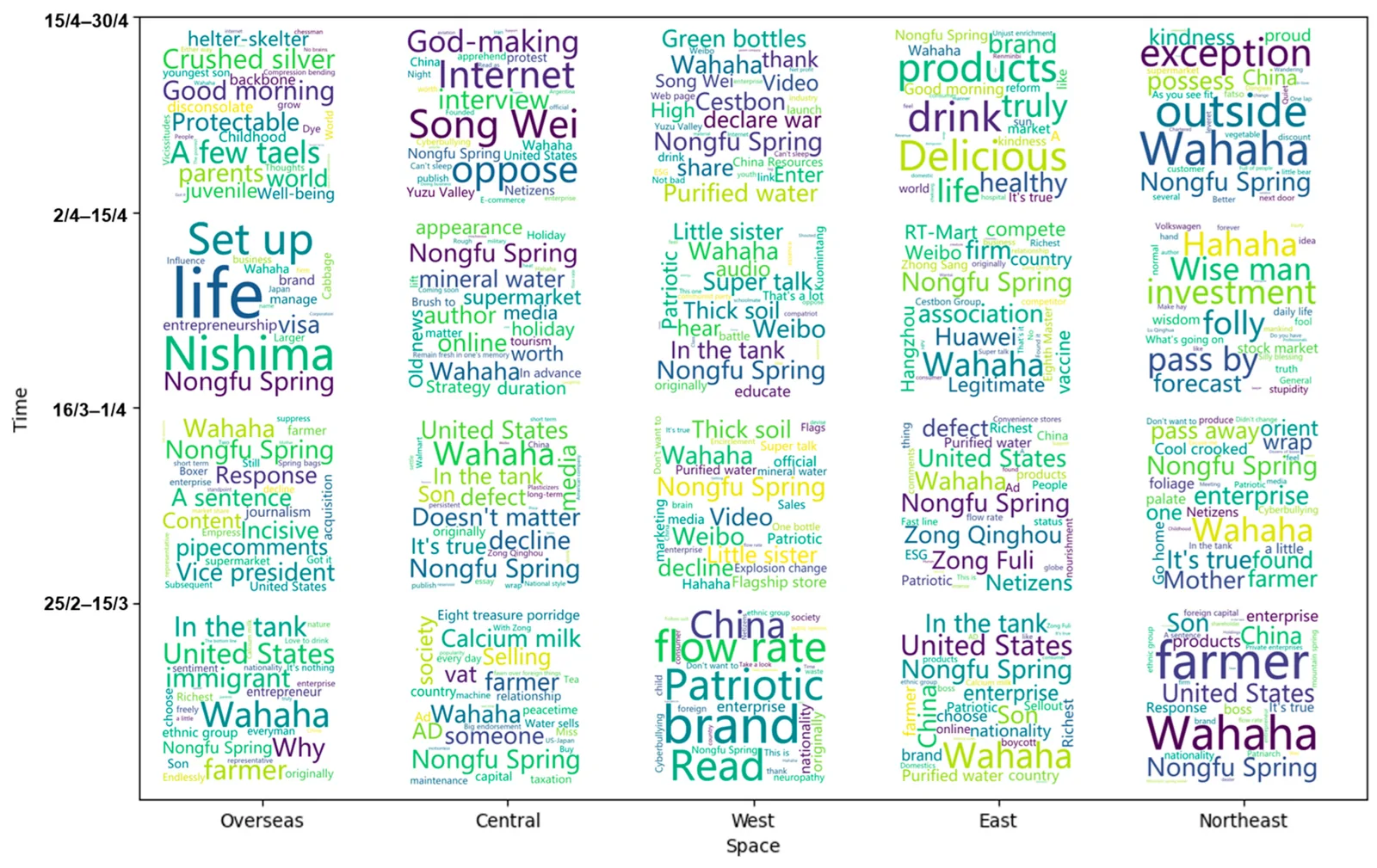
Cross-analysis results between time, space and topic.

The association between phase and region is statistically significant but very weak (χ^2^(12) = 49.41, *p* < 0.001, Cramér’s V = 0.036): regional profiles are broadly similar across phases, and with a sample of this size the significant test result should not be over-read as evidence of substantively large regional differences. Figure 2 displays the joint distribution of time, region, and topic. Between 25 February and 15 March, sampled posts in Central and Eastern China frequently contained keywords such as “Nongfu Spring,” “Wahaha,” “brand,” and “patriotism.” References to “patriotism” and “America” are treated here as discourse labels rather than as indicators of a brand’s actual national orientation. The co-occurrence of Wahaha with support-related terms and of Nongfu Spring with “America” indicates that some users discussed the two brands through identity-related associations; it does not establish that either brand had or lacked any particular affiliation. This phase also contained keywords related to cultural authenticity and brand loyalty. From 16 March to 1 April, the discussion included more market- and leadership-related terms, particularly in the Northeast and Overseas regions, including “market,” “chief,” and “decline.” Posts mentioning Song Wei, founder of YUZU VALLEY, questioned some circulating accusations and called for more cautious interpretation; because the data capture public posts rather than communicators’ intentions, this material is used only as evidence of a more heterogeneous discussion. In April, keywords such as “product,” “competition,” and “investment” became more prominent, indicating greater attention to brand value and market positioning. Posts from Eastern and Central regions more often contained cultural-alignment terms, whereas posts from Northeastern and Overseas regions more often contained terms concerning transparency and competitiveness.

### 4.3. Analysis Results for Time, Space, and Sentiment Categories

**Figure 3:**
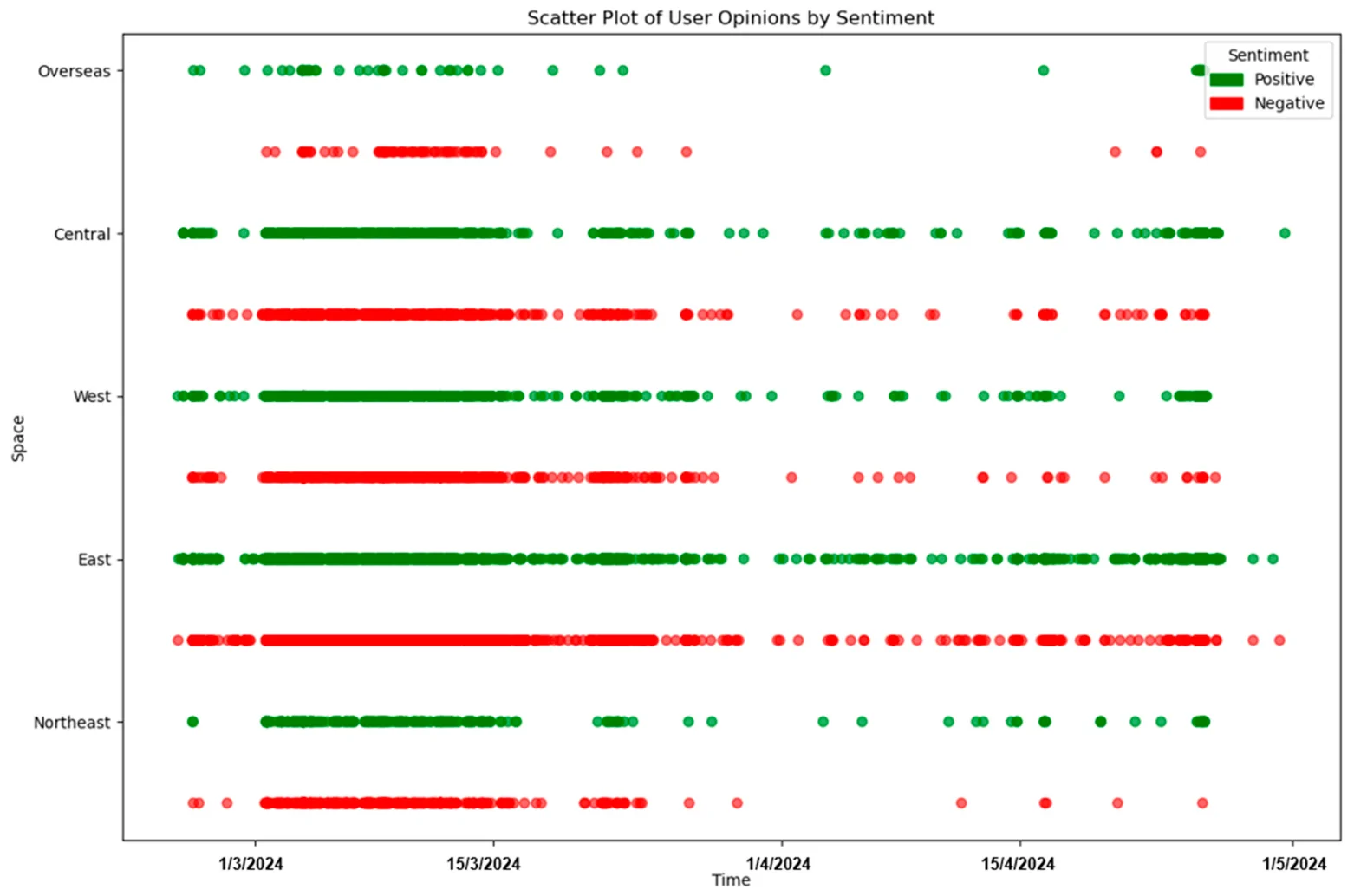
Cross-analysis results between time, space and sentiment.

Sentiment distributions did not differ significantly across regions (χ^2^(4) = 3.99, *p* = 0.407, Cramér’s V = 0.018, N = 12,674), but they differed significantly across phases (χ^2^(3) = 262.93, *p* < 0.001, Cramér’s V = 0.144, N = 12,674). Although the overall Region × Sentiment association was not significant, Figure 3 descriptively shows post-level sentiment classifications across time, space, and sentiment, with positive classifications in green and negative classifications in red. During the early phase (25 February to 15 March), negative classifications were more common in the East and Northeast regions. These classifications describe sentiment expressed in sampled posts and do not establish the factual merits of any claim. Positive classifications were less prevalent but present, particularly in the Central region. As discussion continued from 16 March to 1 April, negative classifications increased in the East and West regions. This pattern coincided with frequent discussion of corporate practices and product integrity, but the observational data cannot establish users’ motivations. The Central region showed mixed classifications. From 2 April to 15 April, both positive and negative classifications appeared in the Central and West regions, indicating continued discussion of brand reputation and consumer trust. Negative classifications remained common in the East, while positive classifications in the Northeast mainly co-occurred with posts expressing support for Wahaha. From 16 April to 30 April, negative classifications were common across most regions, especially the East and Overseas, while the Central and West regions displayed some positive classifications. Overall, Figure 3 describes how post-level sentiment classifications changed across regions and phases; it does not measure offline reputation or behavior.

### 4.4. Analysis Results for Time, User, and Topic Categories

**Figure 4:**
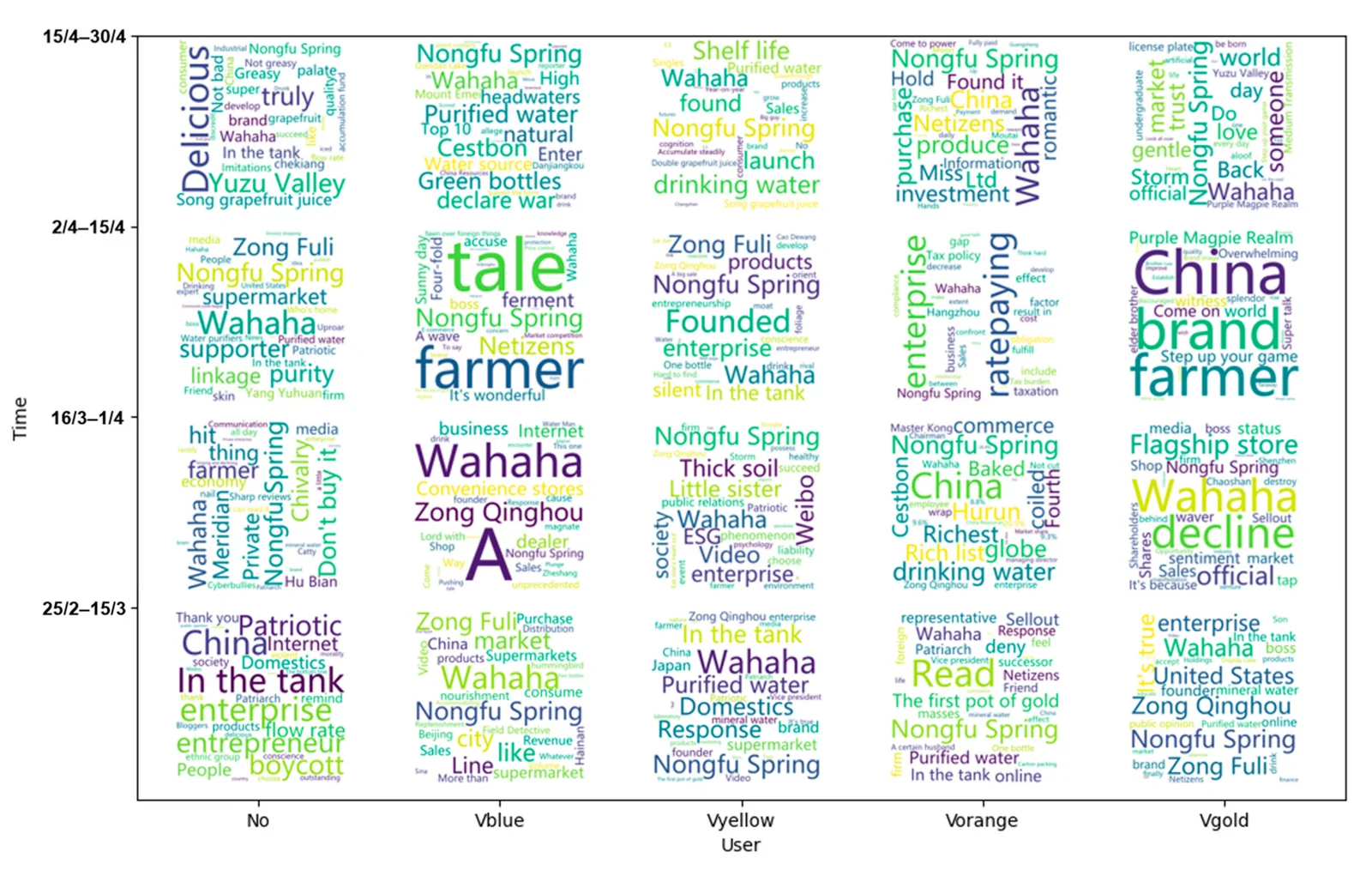
Cross-analysis results between time, user, and topic.

Figure 4 presents the analysis across time, user categories, and topics. From 25 February to 15 March, keywords such as “Nongfu Spring,” “Wahaha,” “support,” and “patriotism” frequently co-occurred among verified users (Vgold and Vblue). These co-occurrences indicate that some sampled posts used national-identity language when discussing Wahaha and associated Nongfu Spring with “America.” They do not, by themselves, verify users’ underlying beliefs or any actual foreign affiliation of the company. Between 16 March and 1 April, terms such as “decline,” “global,” “market,” and “Hu Run” became more prominent, indicating attention to Nongfu Spring’s market performance and international positioning. Verified users, especially Vgold and Vorange accounts, contributed to these discussions; the data do not establish their motives. From April onward, terms such as “purity,” “good,” “YUZU VALLEY,” and “tax” indicate increased attention to product quality and corporate practices. References to “tax” appeared in discussions of financial responsibility and domestic commitment, without allowing an assessment of the factual basis of those discussions.

### 4.5. Analysis Results for Time, User, and Sentiment Categories

**Figure 5:**
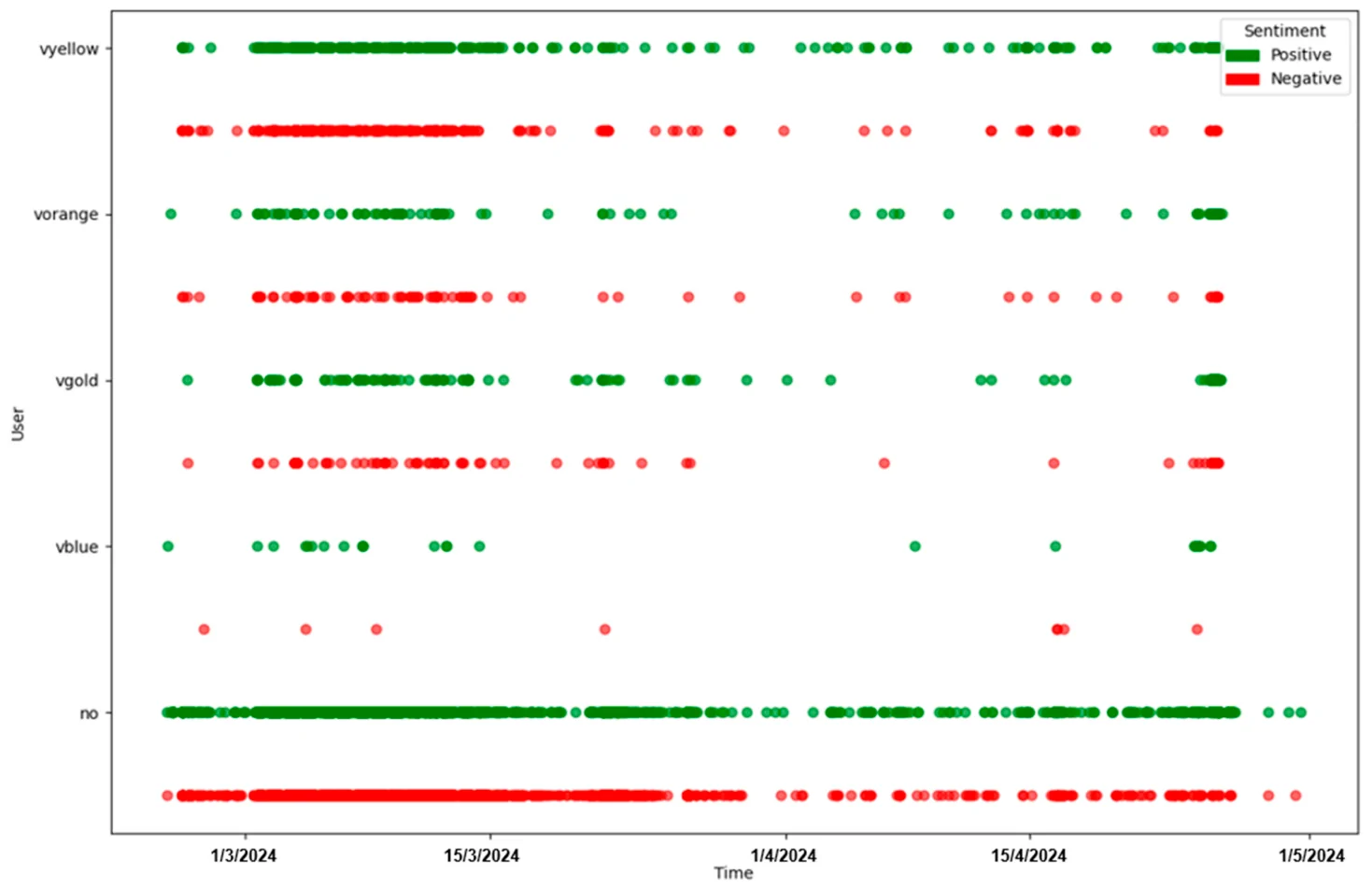
Cross-analysis results between time, user, and sentiment.

Figure 5 provides results regarding time, user categories, and sentiment. From 25 February to 15 March, negative classifications were predominant across all user categories, especially ordinary users and Vblue accounts. This indicates that critical expressions were common during the initial phase, but it does not establish a uniform public response or users’ underlying concerns. From 16 March to 1 April, negative classifications remained common, while positive classifications increased among verified users. Vyellow users showed a balanced mix of positive and negative classifications, and Vgold users exhibited more positive classifications. These differences may reflect varied discussion emphases, such as corporate narratives, historical context, corporate responsibility, or remediation; the observational data cannot determine whether posts reflected strategic communication or other motives. Between 2 April and 15 April, positive classifications increased, particularly among Vgold users, and some posts discussed broader economic implications and potential recovery. Vblue posts remained mixed and included both critical and positive evaluations. From 16 April to 30 April, classifications among ordinary users leaned positive, coinciding with consumer-focused discussions of product quality and reviews. Influential accounts also used brand-loyalty language. Overall, sentiment classifications evolved from predominantly negative to more mixed across the observation period. These findings describe the sampled posts and cannot be generalized to public opinion beyond the dataset. The differences in sentiment across user categories are statistically significant (χ^2^(4) = 125.05, *p* < 0.001, Cramér’s V = 0.099).

### 4.6. Analysis Results for Time, Topic, and Sentiment Categories

**Figure 6:**
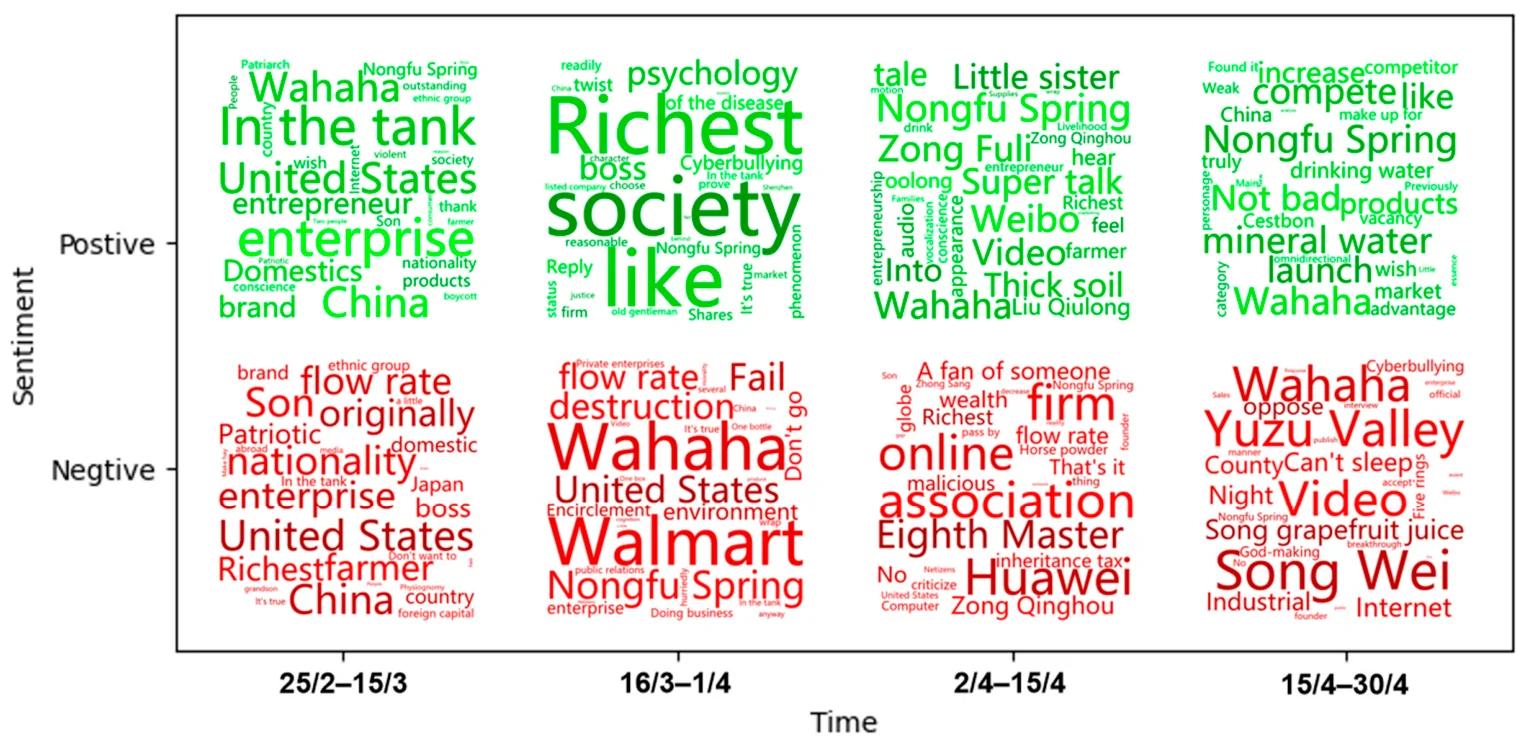
Cross-analysis results between time, topic, and sentiment.

Figure 6 presents the cross-analysis of time, topic, and sentiment across four phases from 25 February to 30 April 2024. In the initial phase (25 February–15 March), keywords such as “support,” “enterprise,” “America,” “China,” and “Wahaha” appeared alongside positive classifications. In sampled posts, Wahaha was sometimes associated with support for domestic brands, whereas Nongfu Spring was sometimes associated with “foreign influence.” These are discourse-level associations, not verified attributes of either company. From 16 March to 1 April, terms such as “collapse,” “psychology,” “chief,” and “social” appeared in negative contexts, indicating that some posts focused on crisis response and leadership. In the third phase (2–15 April), sentiment became more mixed. Positive keywords such as “launch,” “competition,” and brand names suggest renewed attention to product performance and market dynamics, while negative terms such as “company,” “online,” and “Wal-Mart” appeared in discussions of transparency and international positioning. By 16–30 April, the discourse became more diversified: positive classifications centered on product attributes and competition, while negative classifications linked to terms such as “video” and “company” continued to appear. Overall, topic–sentiment associations shifted from identity-related language toward product- and market-related language, although credibility-related concerns remained visible.

### 4.7. Analysis Results for Space, User, and Topic Categories

**Figure 7:**
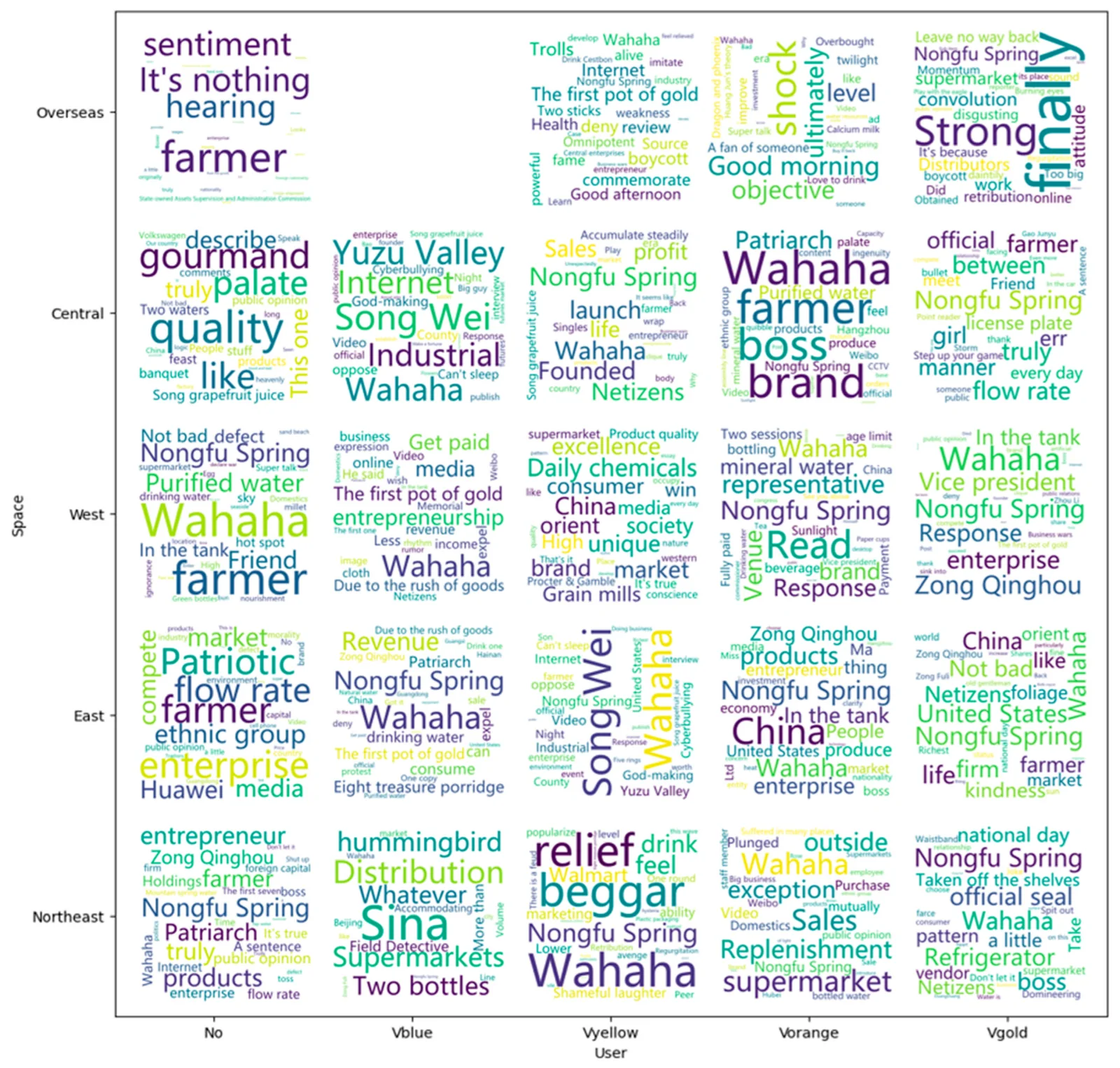
Cross-analysis results between space, user and topic (Note: There are no posts posted overseas by Vblue users).

Figure 7 presents a cross-analysis of region, user category, and topic. In Central and East China, verified users (Vgold and Vblue) frequently co-occurred with the keywords “Nongfu Spring,” “Wahaha,” and “patriotism.” We interpret this as topic co-occurrence within sampled posts, not as a direct measure of users’ national identity. Some posts associated Wahaha with traditional or culturally aligned imagery and Nongfu Spring with “America”; these associations do not establish actual corporate affiliation. In the Northeast, discourse among Vgold and Vorange users contained corporate terms such as “enterprise,” “leadership,” and “market,” indicating discussion of business strategy together with references to national values. Whether this pattern is related to the region’s industrial structure or to local attitudes toward domestic enterprises cannot be determined from the present data. In Western and Overseas regions, both ordinary and verified posts contained keywords such as “network,” “foreign capital,” and “Wahaha,” indicating discussion of corporate influence and global competition, but the data cannot establish users’ motivations.

### 4.8. Analysis Results for Space, User, and Sentiment Categories

**Figure 8:**
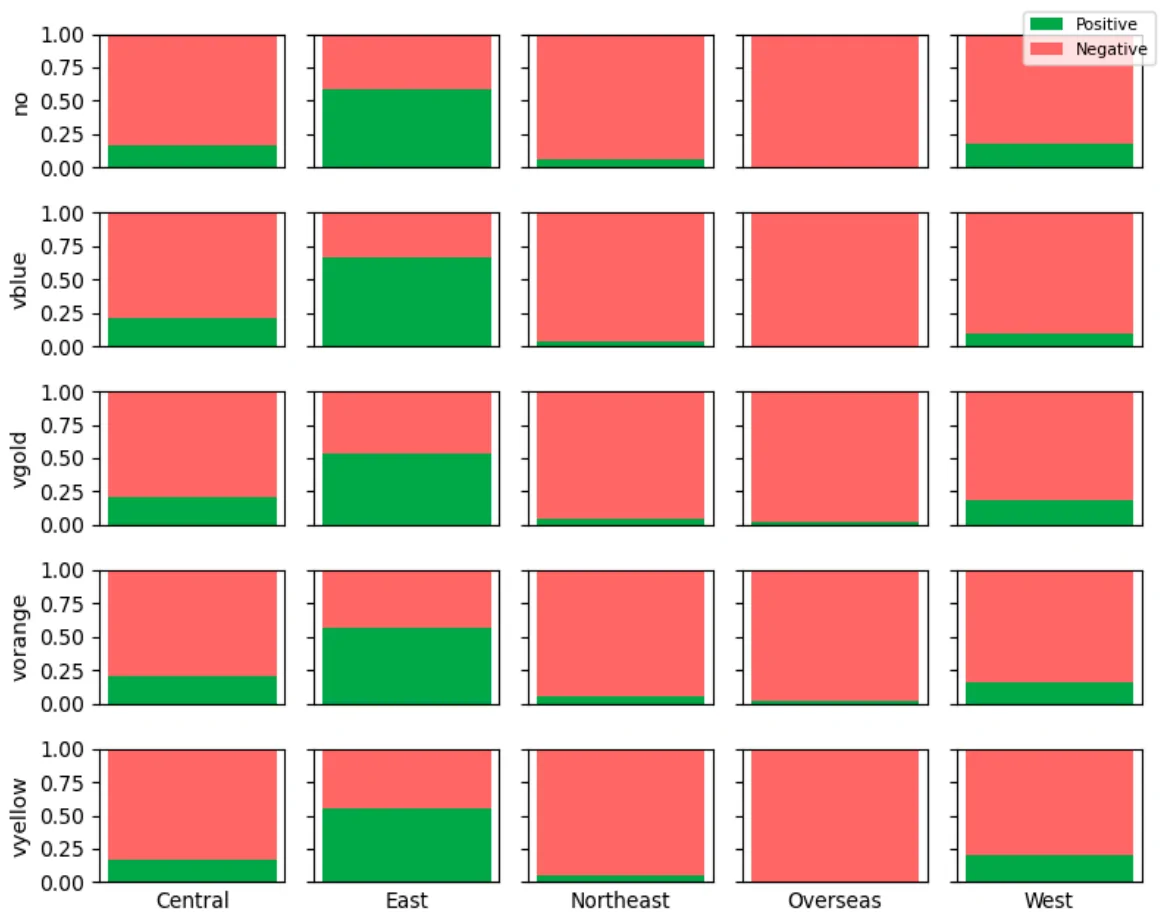
Cross-analysis results between space, user and sentiment.

Figure 8 displays the cross-analysis of space, user, and sentiment. Because the overall Region × Sentiment association was not significant (*p* = 0.407), the patterns in this section are descriptive and exploratory rather than inferential. In the East, a higher proportion of positive classifications is observed across all user categories; the reasons for this regional difference cannot be established with the present data. In the West and Northeast, classifications remain predominantly negative, particularly among ordinary users and Vorange users. The West region’s higher share of negative classifications co-occurred with frequent discussion of the packaging controversy, including posts using the phrase “Japanese-style design elements”; whether identity-related concerns account for this pattern cannot be tested with the present data. The Overseas region shows a higher share of negative classifications across user categories, particularly among Vyellow users. This pattern should not be interpreted as direct evidence about the brand’s international image or consumer behavior, because displayed location is not equivalent to nationality and post-level sentiment does not measure offline attitudes. In contrast, the Central region presents a more balanced distribution, a pattern for which the present data offer no definitive explanation.

### 4.9. Analysis Results for Space, Topic, and Sentiment Categories

**Figure 9:**
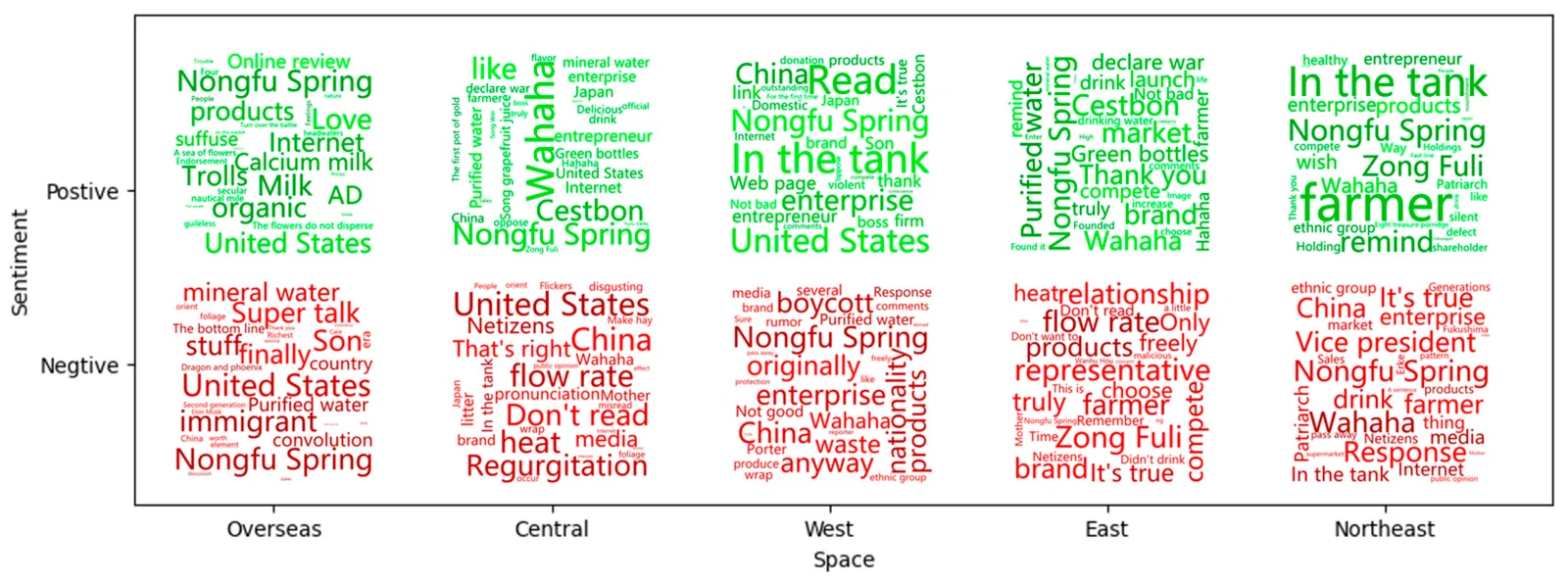
Cross-analysis results between space, topic and sentiment.

Figure 9 describes the co-occurrence of region, topic, and sentiment. As in Section 4.8, the overall Region × Sentiment association was not significant (*p* = 0.407); the patterns described below are therefore descriptive and exploratory rather than inferential. In Central China, positive associations with “Wahaha,” “Nongfu Spring,” and “support” indicate frequent supportive references to domestic brands and the presence of national-identity terms. In the West and Northeast, “Nongfu Spring,” “protest,” and “corporate” appear in negative-sentiment contexts, indicating that some posts questioned brand values or cultural alignment; these patterns do not demonstrate broader distrust across regional populations. Overseas posts associated “Nongfu Spring” with “America” and “immigration” in negative contexts, indicating discussion of global affiliation and national-interest framing. In Eastern China, classifications were mixed: positive terms such as “Yibao” and “market” appeared alongside negative terms such as “traffic” and “relationship.” These descriptive patterns show how sampled posts combined product, identity, and trust-related language; they cannot be generalized to regional populations or treated as evidence of actual corporate conduct.

### 4.10. Analysis Results for User, Topic, and Sentiment Categories

**Figure 10:**
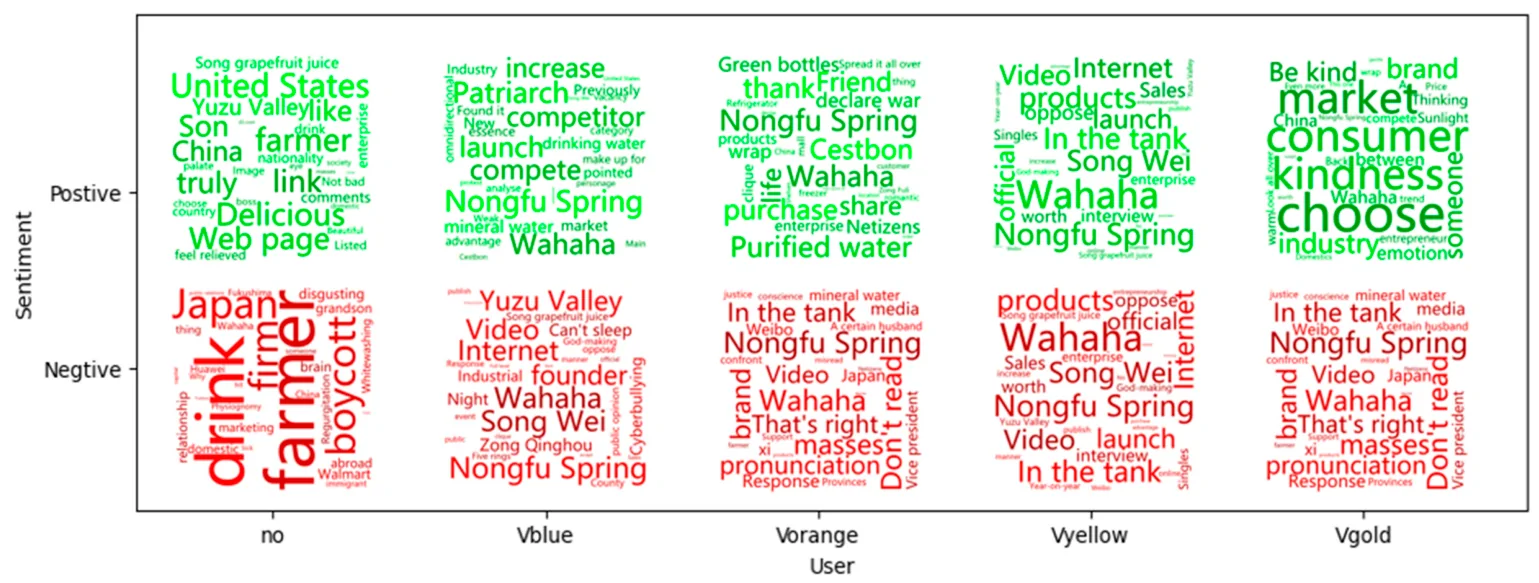
Cross-analysis results between user, topic and sentiment.

The analysis results shown in Figure 10 highlight variation across user categories within the sampled discourse. Verified users (Vgold) combined support for consumer choice and market freedom with criticism of corporate responsibility through negative associations with both Nongfu Spring and Wahaha. This indicates heterogeneity rather than a single stable stance. Vblue users—verified institutional accounts such as media outlets, businesses, and government agencies (*n* = 41 posts; results for this category are indicative only)—expressed positive sentiment toward Wahaha’s legacy and product initiatives and skepticism toward Nongfu Spring’s online image. Ordinary users displayed mixed associations: “America” and “delicious” appeared in positive contexts, while “Nongfu Spring” and its corporate image appeared in negative contexts. These post-level associations do not verify actual foreign ties. Among Vyellow and Vorange posts, positive sentiment centered on Wahaha and national support, while Nongfu Spring was discussed critically in relation to brand authenticity. We do not infer from these patterns that such users generally prefer nationalist brands; rather, the figure shows how different groups of posts combined brand, identity, and product-related terms.

## 5. Conclusions and Discussion

This paper presents a framework for analyzing social media data that emphasizes the interconnections among various elements within the data. We apply this framework to an online controversy involving Nongfu Spring following the death of Zong Qinghou, founder of Wahaha, in February 2024. This case illustrates how the framework can describe the dynamics of sampled social media discourse, including the circulation of identity-related expressions and their co-occurrence with sentiment and brand-related topics. Because the analysis is based on observational Weibo posts, it does not establish effects on offline public perception or consumer behavior. The incident highlights how social media data can be used to map the evolution of controversy-related discourse and corporate reputation talk over time. This phenomenon is echoed in existing literature, such as (Zhou et al., 2021a, 2021b), who discuss the growing influence of social media on brand narratives. Our framework allows for an analysis of these events not as isolated incidents but as part of a broader social context where time, space, user demographics, topics, and sentiments intersect. This perspective aligns with (Xu et al., 2024), who emphasize the need for understanding social media interactions as dynamic and interconnected rather than static.

In addition, the framework we propose encourages methodological flexibility, addressing the limitations often seen in traditional social media studies. Previous studies have highlighted the importance of adaptable frameworks in understanding user interactions and sentiments across digital platforms. For instance, Erfina and Nurul (2023) applied a Naive Bayes algorithm to analyze user sentiments on Twitter regarding AI interactions, underscoring the need for robust, adaptable sentiment analysis tools that can effectively capture public opinion across diverse contexts. In contrast, our approach advocates for a more integrated perspective, allowing researchers to tailor their investigations to specific research objectives, such as user–sentiment cross-analyses, topic evolution, or the policy implications of these cross-analyses. This methodological adaptability is further exemplified in comparative studies, which allows researchers to deepen their understanding of the ways in which cultural factors influence social media discourse and user behavior. Practically, this flexible framework extends across diverse sectors. Businesses and marketers can utilize these insights to design culturally resonant digital campaigns. Moreover, policymakers can benefit from understanding regional sentiments and social trends to create inclusive and responsive policies. Chin et al. demonstrated this by analyzing user-chatbot interactions during the COVID-19 pandemic, identifying significant cultural and emotional differences in user responses to health information (Chin et al., 2023). Such insights are crucial in informing policy decisions that resonate with diverse communities, thereby fostering cross-cultural understanding and cooperation.

Importantly, we clarify that our framework does not necessitate strict adherence in all studies utilizing social media data. Rather, it offers a broad and systematic perspective, allowing researchers to tailor their analytical approach to their specific research objectives. For example, a study concentrated on user–sentiment or user–topic cross-analysis should prioritize those specific associations. Conversely, research aimed at policy analysis may focus on the temporal evolution of topics and sentiments to assess public response and policy effectiveness. Additionally, we recommend that cross-analysis of different elements should be conducted only when substantial differences are observed, ensuring that meaningful conclusions can be drawn. Employing preliminary statistical tests, such as the chi-square test, can help determine whether further analysis of specific relationships is necessary. This recommendation was implemented in the present case study: the cross-analyses interpreted in Section 4 are those for which the pairwise tests among their categorical dimensions indicated statistically significant associations (Table 4); patterns resting on non-significant associations (Section 4.8 and Section 4.9) are explicitly flagged as descriptive and exploratory.

This study has several limitations. First, because the sampling frame combined keywords related to two companies, the dataset blends crisis-oriented posts with tribute posts mourning Zong Qinghou; although the robustness check reported in Section 3.1 indicates that the main temporal and spatial distributions are not driven by the tribute stream, sentiment attributed to the crisis may partly reflect condolence discourse, and future work should separate the two streams through supervised classification. Second, the reliance on a single case and a single platform restricts the generalizability of the findings, and the applicability of the framework to other platforms, languages, and event types remains to be established. Third, identity-related labels such as “patriotism,” “unpatriotic,” and “foreign affiliation” are derived from post content and keyword co-occurrences; they capture discourse framing rather than verified attributes of companies or stable beliefs of users, and future work could triangulate them with independent evidence. Fourth, the spatial dimension relies on Weibo’s displayed IP territory, a coarse-grained, platform-recorded attribute that does not constitute an exact geolocation and may be affected by travel, proxy networks, or other factors; the regional findings therefore describe the distribution of displayed locations and should not be interpreted as the verified geography or residence of users. Future research should encompass a broader spectrum of platforms and case studies to provide a more nuanced understanding of these dynamics. Finally, while our framework addresses key elements, it may overlook other influential factors. Expanding the framework to integrate additional variables could yield richer insights into the complexities of digital media’s role in shaping public opinion and corporate reputation, thereby enhancing the relevance of our findings in light of contemporary challenges.

## Figures and Tables

**Table 1:** Several existing research frameworks for social media data analysis.

	Framework Name	Steps Included in the Framework	Methods Used	Innovations of the Framework
(Zhan et al., 2021)	Social Media Analytic Framework for Retail Pharmacy	1. Data Collection 2. Data Classification and Coding 3. Data Analysis and Information Interpretation	Text Mining, Sentiment Analysis, Social Network Analysis	Uses social media data to highlight most-discussed topics by consumers, identifies key areas for improvement based on negative comments, determines connections amongst important concepts to enhance customer loyalty
(Wukich, 2022)	Structure-Content Framework for Social Media Engagement	1. Data Collection 2. Data Classification 3. Communication Modes Mapping 4. Content Analysis 5. Policy Recommendations	Content Analysis, Case Study	Integrates both communication structure and content goals to characterize government-public social media engagement. Provides a matrix for identifying engagement strategies based on communication modes and content categories, applicable across various contexts.
(Simonofski et al., 2021)	Policy Analytics Framework	1. Agenda setting 2. Formulation 3. Decision 4. Implementation 5. Monitoring	Design Science Research, Data Analysis, Interactive Dashboard Testing	Combines insights from e-participation platforms and social media, supports each step of the policy-making process with appropriate data analytics.
(Agnihotri & John-Mariadoss, 2022)	Integrative Framework for Social Media and B2B Sales	1. Social media usage and performance 2. Measurement issues 3. Future research agenda	Empirical Analysis	Integrates social media use with B2B sales performance at both individual and organizational levels, proposes a multi-method approach for future research.
(Dang et al., 2014)	Dark Web Forum Portal	1. Data Integration Support 2. Search Support 3. Multilingual Translation Support	Complete and Incremental Spidering, full-text indexing, automatic multilingual translation	Addressing multilingual user-generated content. Integration and analysis of content from different social media sites.
(Sivarajah et al., 2020)	Social Media Analytics Framework for B2B Marketing	1. Identifying, Tracking, and Acquiring Data 2. Preprocessing Transformations 3. Analysis Techniques	Social media data analysis, data scraping, big data analysis	Emphasis on strategic implications of social media analytics for B2B marketing. Focus on post-analysis stage.
(Jimenez-Marquez et al., 2019)	Data Integration and Big Data Analytics Framework	1. Data Entry 2. Information Retrieval, 3. Data Preparation, 4. Structure and Modeling, 5. Machine Learning Model	Machine learning and Natural Language Processing techniques, sentiment analysis, recommender systems, user reviews analysis	Two-stage process (non- big data environment for initial analysis and big data environment for advanced analysis), and adaptable to new domains and algorithms.
(Yin et al., 2022)	Social Media and Spatial Framework	1. Manual annotation, 2. Pre-processing 3. Vectorization 4. Classification 5. Reply network construction 6. Community detection in reply networks 7. Hybrid space networks analysis	Sentiment analysis, social network analysis, hybrid space networks	Examine the information propagation between cyber, relational, and physical spaces.
(Déom et al., 2023)	5C Model	1. Confidence 2. Complacency 3. Constraints 4. Calculation 5. Collective responsibility	Mixed-methods approach, qualitative discourse and network analysis, rapid appraisal design	Utilization of mixed-methods for rapid analysis, integration of social media monitoring software, and in-depth use of text network analysis software for betweenness centrality analysis.
(Kolajo et al., 2020)	Social Media Feed Pre-processing Framework	1. Data collection 2. Data cleaning 3. Data transformation 4. Slang classification 5. Semantic analysis using Integrated Knowledge Base and adapted Lesk algorithm	Data cleaning and transformation, slang classification, semantic analysis using Integrated Knowledge Base, adapted Lesk algorithm for disambiguation	The integration of local knowledge sources, such as Naijalingo, Urban Dictionary, and internet slang, enhances the disambiguation of slang, abbreviations, and acronyms, leading to improved accuracy in sentiment analysis and event detection using local context.
(Papafitsoros et al., 2023)	Social Media Interaction Model	1. Collection of social media records 2. Identification of interaction events 3. Analysis of social media activity 4. Temporal comparison of viewing pressures	Social media data mining, empirical validation using Instagram data, analysis of temporal changes in human–animal interactions, validation through recorded visitation numbers	Social media data is used to infer real-time wildlife viewing pressures, automate the detection of human–wildlife interactions, and validate models using empirical data from social media.
(Thomaz et al., 2017)	Content Mining Framework in Social Media	1. Define objectives, domains, and other definitions 2. Define technology support required 3. Data collection strategies and execution 4. Data cleaning and treatment 5. Data mining 6. Results interpretation and evaluation 7. Results visualization	Text mining, data conditioning, predictive analysis, data cleaning and processing, ontology-based relevant term identification, manual and automatic categorization, sentiment analysis.	This study combines text mining and ontology-based methods, enables real-time reaction through a self-response system, and offers a comprehensive solution for handling large, noisy, unstructured, and dynamic social media data.
(Chung & Lai, 2023)	Temporal Graph Framework	1. Collect social network data 2. Extract temporal bipartite networks 3. Model behavioral effects 4. Simulate and predict SMN activities 5. Model configuration and evaluation 6. Performance measures	HDBSCAN clustering, feedforward-backpropagation neural network, Markov Chains, Tensor Factorization, Conformity, Recency-Primacy, Trend, Periodic models	This approach integrates social network concepts and time-series methods, employs dynamic network modeling, and introduces a novel application in cryptocurrency social media networks (SMNs) for multi-faceted prediction of SMN activities.
(Qin et al., 2024)	Collaborative Emergency Decision-Making Framework	1. Extract keywords from social media data 2. Define 2-tuple emergency attributes 3. Perform sentiment analysis 4. Collaborative decision-making involving public and expert opinions 5. Sensitivity analysis 6. Comparative study against existing models	Sentiment analysis, multi-phase collaborative model, sensitivity analysis	This approach combines public opinion and expert knowledge, utilizes XLNet-Att for sentiment analysis, and implements a multi-phase collaborative model, which is applied in the real-world case of the Southern China flood disaster.
(M. A. Khan & AlGhamdi, 2024)	Customized Deep Learning Framework	1. Input Layer 2. Word Embedding Layer 3. LSTM Layer 4. Fully Connected Layer 5. Softmax Layer 6. Customized Classification Layer	Long Short-Term Memory, K-Nearest Neighbors, Linear Discriminant Analysis, Customized SSE Loss Function.	Enhanced classification accuracy for social media posts related to Hajj and Umrah services using SSE loss function instead of cross-entropy loss.
(Singh & Verma, 2022)	Effective Parallel Processing Social Media Analytics Framework	1. Streaming and Search API 2. Message Broker 3. Pre-processing Block 4. Database and Cache Management 5. Analytics Block 6. Visualization	Redis, MongoDB, AYLIEN API, Parallel Processing.	Lightweight, fast, and robust framework for real-time processing of social media posts with parallel processing and message brokers.
(Manroop et al., 2022)	Applicant Reactions Framework	1. Data Collection 2. Pre-processing 3. Sentiment Analysis 4. Bias and Adverse Impact Analysis 5. Decision Making	Machine learning	Framework specifically designed to handle applicant reactions to social media assessments with considerations for legality, reliability, and job relevance.
(Zachlod et al., 2022)	Social Media Analytics Framework	1. Discovery 2. Tracking 3. Preparation 4. Analysis	Statistical Analysis, Social Network Analysis, Sentiment Analysis, Content Analysis, Trend Analysis.	Extended existing framework by adding the Discovery step to address challenges in event and topic detection.
(Y. Chen et al., 2022)	Social Media Representation Bias Framework	1. Setting Representation Bias Benchmark 2. Modeling Sampling Uncertainty 3. Quantifying Representation Bias	Bayesian-based modeling, demographic prediction, statistical analysis	Systematic approach to address and quantify social media representation bias in disaster scenarios.
(Mahyoob et al., 2022)	Social Media Analytics Framework for SARS-CoV-2 Omicron	1. Data Collection 2. Data Cleaning 3. Sentiment Analysis 4. Visualization	SentiStrength, Voyant-tools	Combining sentiment analysis with social media data to analyze public emotions towards Omicron.
(Pilař et al., 2021)	Social Media Analysis Based on Hashtag Research	1. Data Cleaning 2. Data Integration 3. Data Transformation 4. Data Mining 5. Data Evaluation 6. Network Visualization	Hashtag Matcher, Eigenvector Centrality, Degree Centrality	Focus on hashtags to provide insights for strategic marketing and management, identification of community clusters in social media.
(M. L. Khan et al., 2021)	Public Engagement Model	1. Content Analysis 2. Theme Identification 3. Engagement Analysis	SentiStrength, NVivo, Text Analytics	Highlighting key factors of public engagement in digital diplomacy such as self-disclosure, positive attitude, and inquisitiveness.
(Jindal & Aron, 2021)	Visual-Textual Sentiment Analysis Framework	1. Visual Analysis: Preprocessing, Feature 2. Extraction, Feature Selection using ICOA 3. Textual Analysis: Preprocessing, Word Embedding, Feature Extraction 4. Polarity Classification using BMMCA-DenseNet	Visual Analysis: Image Denoising, Image Resizing, Image Enhancement, RGB Color Histogram, GLCM, SIFT, Emoticon and Non-emoticon Features; Textual Analysis: Tokenization, Stop Word Removal, Lemmatization, URL Removal, Adaptive ELMo	Utilization of both visual and textual data for sentiment analysis, improved feature selection using ICOA, integration of BMMCA-DenseNet for polarity classification.
(Sheikhattar & Mansouri, 2024)	Topic Mapping-based Knowledge Discovery System	1. Data Gathering 2. Pre-processing: Sentence Splitting 3. Tokenization 4. Word Embedding Model Generation 5. Normalize Corpus 6. Feature Selection 7. Topic Extraction 8. Expand Topic Map 9. Enrich Query 10. Extract Knowledge Units 11. Evaluate Knowledge Units	TF-IDF, NMF for topic extraction, Word2Vec for word embedding, user-system interaction for iterative knowledge extraction	Application of topic mapping for unstructured risk data, interactive user-system approach for iterative knowledge extraction, utilization of text mining and thematic analysis for supply chain risk data.
(X. Han et al., 2024)	Trajectory-based Grid Semantic Cube	1. Gridding of spatial data 2. Construction of behavioral trajectory cube 3. TGS-weighted similarity measurement 4. Spectral clustering 5. PrefixSpan for semantic pattern mining	Spectral clustering, PrefixSpan algorithm, EDR and LCSS similarity measures	Integration of spatial-temporal-semantic features for public behavior analysis, Enhanced accuracy with TGS-weighted similarity measure, Visualization of behavior trajectory cube.
(Ali et al., 2022)	BSDaaS (Big Social Data as a Service)	1. Data collection 2. Data preprocessing 3. Location extraction 4. Data analysis 5. Service composition plan generation	Graph-Planning algorithm, Sentiment analysis, Data quality model, Service-oriented architecture	Dynamic assessment of social media data quality, Multi-pipeline service composition, Quality-driven service selection.

**Table 2:** Matrix considering relationships between pairs of elements of social media data.

	Time	Space	User	Topic	Sentiment
Time	/	Temporal Patterns by Region	User Behavior Over Time	Topic Evolution Across Time	Temporal Sentiment Fluctuations
		Identification of peak times by region for higher engagement	Analysis of posting frequency across user demographics	Seasonal or cyclic topics and their recurring engagement patterns	Tracking of emotional peaks during significant periods (e.g., crisis sentiment surges)
		Time-specific trends in certain areas during crises or events	Identification of high-activity periods for different user types	Real-time tracking of emerging topics during crises	Identification of sentiment trends across time (e.g., mood shifts from positive to negative during prolonged debates)
		Evolution of regional engagement across time (e.g., shifts from interest to apathy over prolonged events)	Tracking user loyalty and re-engagement over time	Decline or growth of specific topics over time as influenced by events	Correlation between time of day and sentiment in user engagement
Space	/	/	Regional User Demographics and Preferences	Regional Trends in Topic Engagement	Geographic Sentiment Insights
			Analysis of local user demographics and preferences for location-based customization	Mapping of location-specific interests and their popularity	Analysis of sentiment variations by region in response to events
			Understanding regional differences in engagement	Cultural significance in topics across regions	Identification of “sentiment hotspots” where emotions are consistently high
			Community formation based on regional connections	Localization of certain topics due to regional relevance or events	Culturally driven sentiment differences and regional emotional responses
User	/	/	/	User-Topic Segmentation and Preferences	User Sentiment Profiles
				Mapping user interests to categorize communities and preferred topics	Understanding how different user types react emotionally (e.g., sentiment across age or interest groups)
				Analysis of topics’ appeal to specific user demographics	Sentiment variation by user demographics, revealing group-based emotional responses
				Insights into theme-based segmentation within user networks	Patterns in engagement based on sentiment-driven content
Topic	/	/	/	/	Topic-Specific Sentiment Trends
					Analysis of emotional tone variations across different topics
					Tracking of sentiment over time within controversial or polarizing topics
					Understanding topic-driven sentiment clusters within discussions
Sentiment	/	/	/	/	/

**Table 3:** Descriptive statistics of the final dataset (N = 12,674).

Dimension	Category	Posts	Share (%)
Region	East	7348	57.9
Region	Central	2137	16.9
Region	West	2274	17.9
Region	Northeast	704	5.6
Region	Overseas	211	1.7
User category	Ordinary	10,883	85.9
User category	Vyellow	1122	8.8
User category	Vgold	328	2.6
User category	Vorange	300	2.4
User category	Vblue	41	0.3
Phase	Phase 1 (25 February–15 March)	11,028	87.0
Phase	Phase 2 (16 March–1 April)	808	6.4
Phase	Phase 3 (2–15 April)	157	1.2
Phase	Phase 4 (16–30 April)	681	5.4

**Table 4:** Chi-square tests of independence for the dimension intersections.

Intersection	χ^2^	df	N	*p*	Cramér’s V
Phase × Region	49.41	12	12,674	<0.001	0.036
Phase × User	2006.45	12	12,674	<0.001	0.230
Region × User	38.99	16	12,674	0.002	0.028
Phase × Sentiment	262.93	3	12,674	<0.001	0.144
Region × Sentiment	3.99	4	12,674	0.407	0.018
User × Sentiment	125.05	4	12,674	<0.001	0.099

Note: *p*-values for Phase × User and Region × User are Monte Carlo *p*-values based on 20,000 replicates; all other *p*-values are asymptotic.

## Data Availability

The data used to support the findings of this study are available from the corresponding author upon request.
